# Effects of process factors on performances of liquid membrane-based transfer of indole-3-acetic acid

**DOI:** 10.1038/s41598-021-02876-x

**Published:** 2021-12-06

**Authors:** Ioana Diaconu, Oana Cristina Pârvulescu, Sorina Laura Topală, Tănase Dobre

**Affiliations:** 1 Department of Analytical Chemistry and Environmental Engineering, University POLITEHNICA of Bucharest, 1-6 Gheorghe Polizu, 011061 Bucharest, Romania; 2 Department of Chemical and Biochemical Engineering, University POLITEHNICA of Bucharest, 1-6 Gheorghe Polizu, 011061 Bucharest, Romania

**Keywords:** Chemical engineering, Statistics

## Abstract

The paper has aimed at studying the transfer of indole 3-acetic acid (*IAA*) from a feed aqueous solution to a stripping aqueous solution of *NaOH* using a chloroform bulk liquid membrane and trioctylamine (*TOA*) as a ligand (*L*). Initial molar concentrations of *IAA* in the feed phase, *c*_*IAA,F*0_ (10^–4^–10^–3 ^kmol/m^3^), of *TOA* in the membrane phase, *c*_*L,M*0_ (10^–2^ and 10^–1^ kmol/m^3^), and of *NaOH* in the stripping phase, *c*_*NaOH,S*0_ (10^–2^ and 1 kmol/m^3^), were selected as process factors. Their effects on the final values of *IAA* concentration in the feed phase (*c*_*IAA,Ff*_) and stripping solution (*c*_*IAA,Sf*_), extraction efficiency (*E*_*F*_), distribution coefficient (*K*_*D*_), and recovery efficiency (*E*_*R*_) were quantified using multiple regression equations. Regression coefficients were determined from experimental data, i.e*.*, *c*_*IAA,Ff,ex*_ = 0.02–1 × 10^–4^ kmol/m^3^, *c*_*IAA,Sf,ex*_ = 0.22–2.58 × 10^–3^ kmol/m^3^, *E*_*F,ex*_ = 90.0–97.9%, *K*_*D,ex*_ = 9.0–46.6, and *E*_*R,ex*_ = 66.5–94.2%. It was found that *c*_*IAA,F*0_ had the most significant positive effect on *c*_*IAA,Ff*_ and *c*_*IAA,Sf*_, whereas *c*_*NaOH,S*0_ had a major positive effect on *E*_*F*_, *K*_*D*_, and *E*_*R*_. A deterministic model based on mass transfer of *IAA* was developed and its parameters, i.e*.*, mass transfer coefficient of *IAA-L* complex in the liquid membrane (0.82–11.5 × 10^–7^ m/s) and extraction constant (1033.9–1779.7 m^3^/kmol), were regressed from experimental data. The effect of *c*_*L,M*0_ on both parameters was significant.

## Introduction

Phytohormones are a group of natural substances found in plants with the role of regulating plant growth and development. The main classes of phytohormones are auxins, abscisic acids, gibberellins, ethylene, and cytokinins^1,2^. Indole 3-acetic acid (*IAA*) is an auxin which has an important role in various plant physiological processes, e.g., cell elongation, division, and differentiation, flowering, fruit development^1–7^.

*IAA*-based biofertilizers and biostimulants can heavily improve soil fertility and crop production^5^. Phytohormones are synthesized by plants at very low levels (0.1–50 ng/g fresh weight basis) and the plants contain many other compounds with similar structure^1,2^. Accordingly, finding simple, fast, and efficient methods for their separation and analysis is a very important task as well as a challenge.

Several analytical techniques were developed for phytohormone analysis, e.g., gas chromatography (GC), GC coupled with mass spectrometry (GC–MS), spectrophotometry, high performance liquid chromatography (HPLC), HPLC coupled with mass spectrometry (HPLC–MS), ultraviolet (HPLC–UV) or fluorescence detection (HPLC-FD)^1,2,4–6,8,9^. A preliminary stage of sample pretreatment before the instrumental analysis is commonly necessary to purify and concentrate the target phytohormone(s)^1,9^. Due to the low phytohormone concentration in the plants as well as to the presence of other compounds with a similar structure, sample pretreatment is a critical stage^1,2^. In addition to the significant loss of analyte mass, this stage can be time-consuming and introduce errors in subsequent quantification^6^.

Solid phase extraction (SPE) is the most common pretreatment technique applied before the quantification of phytohormone content by instrumental analysis^1^. SPE is simple, fast, but commonly has a low selectivity as well as it requires a large solvent volume, resulting in a limited enrichment factor^1,9^. Solid phase microextraction (SPME) allows simultaneous extraction and concentration of analytes^8,9^. SPME is characterized by a low solvent consumption and a high enrichment factor, but requires specialized equipment and usually has low selectivity and efficiency^1,9^.

Selection of efficient methods for the separation and purification of *IAA* carboxylic acid has become a major research aim. Various techniques for the separation of carboxylic acids from aqueous solutions, including liquid–liquid extraction, adsorption, nanofiltration, ultrafiltration, ion exchange, electrodialysis, reverse osmosis, distillation, liquid membrane separation (LMS), have been intensively studied^10–16^. LMS has a series of advantages, e.g., fast and selective separation of target compounds, high transport efficiency, recovery of different compounds with low concentrations, low cost compared to other methods of separation, simple handling, easy to scale up^10–13,17–20^. Accordingly, LMS could be an efficient sample pretreatment applied before instrumental analysis of *IAA*.

Liquid membranes are semi-permeable barriers that separate two aqueous phases, i.e., feed (source or donor) and stripping (receiving or acceptor) phases. Techniques based on bulk liquid membranes (BLMs), emulsion liquid membranes (ELMs), and supported liquid membranes (SLMs) have been widely used to separate organic compounds and metal ions from aqueous solutions^21–24^. BLM-based separation technique is the most simple and efficient among them^20^. BLMs have been extensively used in the separation of carboxylic acids, e.g., formic, acetic, propionic, butyric, levulinic acids^11–13^.

Solute transfer through systems containing liquid membranes can be significantly enhanced by adding different carriers (ligands or extraction reagents) in the liquid membrane. Mass transfer assisted by a carrier takes place as follows: (1) the carrier reacts with the target compound (solute) at the interface between feed phase and membrane phase forming a chemical complex; (2) solute-carrier complex diffuses through liquid membrane and reaches the interface between membrane and stripping phase, where the decomplexation occurs; (3) the solute is released into the stripping phase, whereas the carrier diffuses back through membrane^10–12,23,25,26^. A suitable carrier facilitates the solute extraction (from feed phase) and transport (through membrane phase) as well as determines its purification^24^.

Neutral, acidic (anionic), and basic (cationic) carriers have been extensively used in different applications both on a laboratory and industrial scale^24^. Tri-n-butyl-phosphate (*TBP*) and tri-n-octyl phosphine oxide (*TOPO*) are neutral carriers widely applied to transport organic compounds (e.g., formic, acetic, propionic, butyric, acrylic, levulinic, hippuric, and mandelic acids, vanillin, catechol) and metal ions through liquid membranes^10,11,24,27^. Acidic carriers such as di-(2-ethylhexyl)phosphoric acid (*D*2*EHPA*), bis(2,4,4-trimethylpentyl)phosphinic acid (Cyanex 272), and derivatives of Cyanex 272 have been used to extract and transport amino acids, peptides, and metal ions^24,28^. Trioctylamine (*TOA*) and N-methyl-N,N,N-trioctylammonium chloride (Aliquat 336) are basic carriers commonly involved in liquid membrane-based separations^24^. *TOA* has been extensively applied to separate carboxylic acids (e.g., acetic, propionic, and lactic acids) and metals, whereas Aliquat 336 to extract phenolic compounds, antibiotics, and metals^13,24,29^. Selecting suitable liquid membranes and carriers, BLM-based separation could be successfully applied to isolate and concentrate *IAA* in a single step.

Mathematical modelling is an effective tool used to predict the performances of liquid membrane-based separation^11,17–20,28,30,31^. Process performances depend on different factors, e.g., type and initial concentration of separating species in the feed phase, type of organic solvent in the membrane phase, type and concentration of carrier in the membrane phase, type and concentration of stripping agent in the stripping phase, type of separation equipment, stirring speed, temperature, pH of feed and stripping phases, volumes of feed, membrane, and stripping phases and their contact surface areas.

The transport of *IAA* through a chloroform BLM using *TOA* as a basic (cationic) carrier was studied in this paper. Statistical models based on a 2^3^ factorial plan and a deterministic model based on mass transfer of *IAA* were used to predict the process performances under different operation conditions. Chloroform is widely used as BLM^21,23,24,32^. It has a lower viscosity (*η* = 0.58 cP) than other solvents, e.g*.*, 1,2-dichloroethane (*η* = 0.73 cP), nitrobenzene (*η* = 1.62 cP), resulting in a faster mass transfer^21^. *TOA* carrier can heavily improve the separation efficiency. *TBP*, *TOPO*, and *TOA* carriers and chloroform BLM were used in a previous study to separate *IAA* from dilute aqueous solutions^32^. Separation efficiency was higher for *TOA* carrier, due to stronger donor–acceptor interactions between *IAA* and *TOA*, as opposed to weaker hydrogen bonds between *IAA* and the other two carriers.

## Materials and methods

### Materials

*IAA*, chloroform, *TOA*, and *NaOH*, which were provided by Merck (Germany), were analytical grade reagents used without further purification. Three-phase system involved in the mass transfer process consists of: (1) a feed (*F*) aqueous solution of *IAA*; (2) a membrane (*M*) phase consisting of a chloroform BLM and *TOA* as a ligand (*L*); (3) a stripping (*S*) aqueous solution of *NaOH*.

### Experimental setup and process parameters

**Figure 1:**
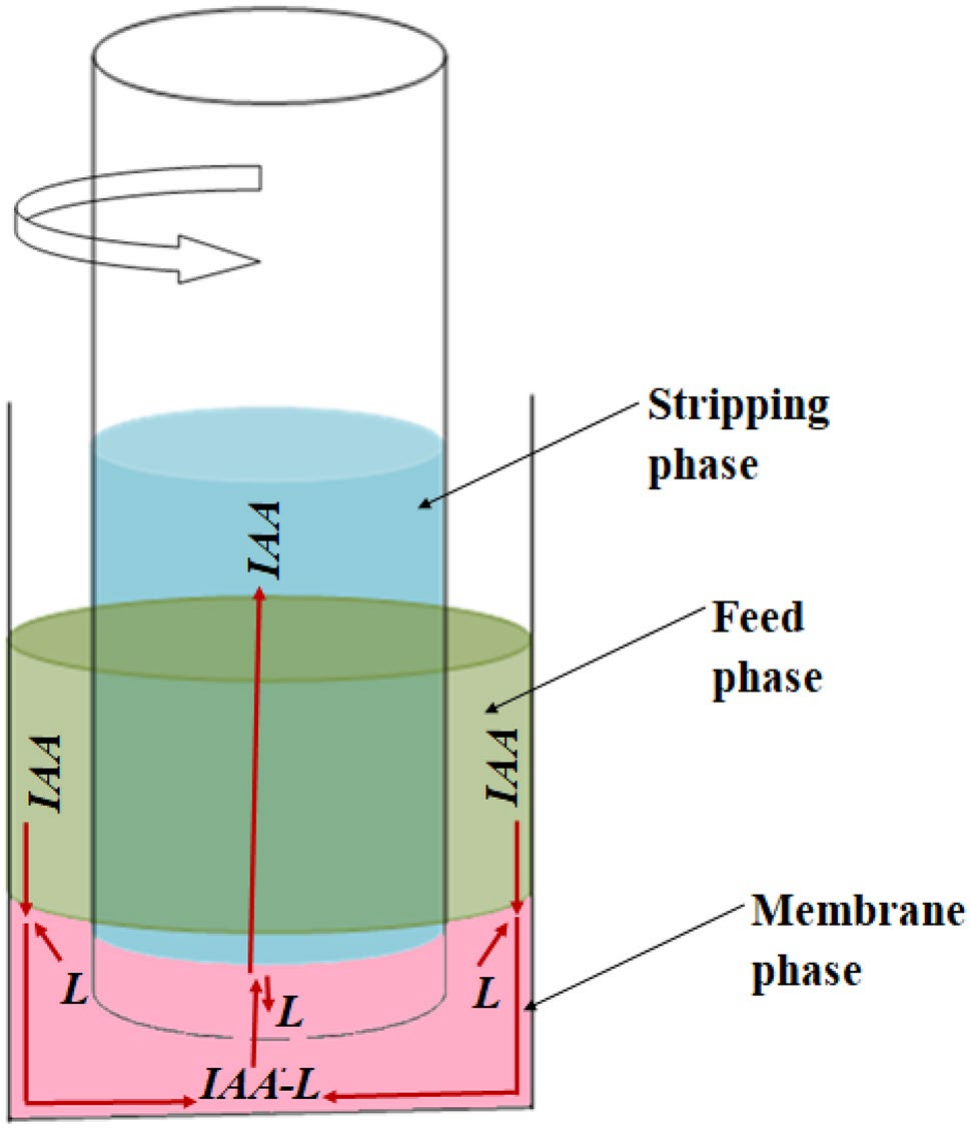
Scheme of experimental setup.

A scheme of experimental setup used to study the transfer of *IAA* from the feed phase to stripping phase is shown in Fig. 1. The tube in tube setup consists of an outer glass tube, containing the feed solution (at the upper part) and membrane phase (at the bottom part), and an inner glass tube, containing the stripping solution^32–34^. The internal diameter of outer tube was *D*_*in*_ = 0.042 m, whereas the external and internal diameters of inner tube were *d* = 0.021 m and *d*_*in*_ = 0.019 m, respectively. The values of phase volumes were *V*_*F*_ = 20 × 10^–6^ m^3^, *V*_*M*_ = 50 × 10^–6^ m^3^, and *V*_*S*_ = 7 × 10^–6^ m^3^.

According to the schematic representation in Fig. 1, the mass transfer process in the experimental setup occurs as follows: (1) *IAA* diffuses through *F* phase towards interface between *F* and *M* phases; (2) *TOA* (*L*) diffuses through *M* phase towards *F-M* interface; (3) *IAA* reacts with *L* at the interface forming the *IAA-L* complex; (4) *IAA-L* complex diffuses through *M* phase and reaches the interface between *M* and *S* phases, where the decomplexation takes place; (5) *IAA* is released into *S* phase and *L* diffuses back through *M* phase.

Initial molar concentrations of *IAA*, *TOA*, and *NaOH* in *j* (*F*, *M*, *S*) phase, namely *c*_*IAA,F*0_, *c*_*L,M*0_, and *c*_*NaOH,S*0_, were selected as process independent variables (factors). Twenty experimental runs were conducted at different levels of *c*_*IAA,F*0_ (10^–4^–10^–3^ kmol/m^3^), *c*_*L,M*0_ (10^–2^ and 10^–1^ kmol/m^3^), and *c*_*NaOH,S*0_ (10^–2^ and 1 kmol/m^3^). Each experiment was performed for 4 h, at ambient temperature, under mechanical stirring (200 rpm) of inner tube.

Final (*f*) values of molar concentration of *IAA* in the feed (*c*_*IAA,Ff,ex*_) and stripping solution (*c*_*IAA,Sf,ex*_), corresponding to *τ*_*f*_ = 14,400 s, were determined experimentally using a LAMBDA 750 spectrophotometer (PerkinElmer, USA). Extraction efficiency (*E*_*F,ex*_), distribution coefficient (*K*_*D,ex*_), and recovery efficiency (*E*_*R,ex*_) are defined by Eqs. (1)–(3)^10–12,18,30^.

1 $$E_{F,ex} = 100\frac{{c_{IAA,F0} - c_{IAA,Ff,ex} }}{{c_{IAA,F0} }}$$

2 $$K_{D,ex} = \frac{{c_{IAA,F0} - c_{IAA,Ff,ex} }}{{c_{IAA,Ff,ex} }}$$

3 $$E_{R,ex} = 100\frac{{c_{IAA,Sf,ex} }}{{c_{IAA,F0} }}$$

### Statistical analysis

Multiple regression analysis and correlation analysis were performed using XLSTAT 2019.1 (Excel).

### Mathematical modelling

#### Statistical models

The effects of process factors (*c*_*IAA,F*0_, *c*_*NaOH,S*0_, and *c*_*L,M*0_) on dependent variables (responses), i.e., *c*_*IAA,Ff*_, *c*_*IAA,Sf*_, *E*_*F*_, *K*_*D*_, and *E*_*R*_, were quantified using statistical models based on a 2^3^ factorial plan^35,36^. According to a 2^3^ factorial plan, 8 experimental runs (1–8 in Table 1) were conducted at 2 levels (inferior and superior) of process factors. Dimensionless values of process factors are given by Eqs. (4)–(6), where *c*_*IAA,F*0*,cp*_ = 0.00055 kmol/m^3^, *c*_*NaOH,S*0*,cp*_ = 0.505 kmol/m^3^, and *c*_*L,M*0*,cp*_ = 0.055 kmol/m^3^ are centre-points.

4 $$x_{1} = \frac{{c_{IAA,F0} - 0.00055}}{0.00045}$$

5 $$x_{2} = \frac{{c_{NaOH,S0} - 0.505}}{0.495}$$

6 $$x_{3} = \frac{{c_{L,M0} - 0.055}}{0.045}$$

**Table 1:** Experimentation matrix for 2^3^ factorial experiment.

Run	*c*_*IAA,F*0_ × 10^3^ (kmol/m^3^)	*c*_*NaOH,S*0_ × 10^3^ (kmol/m^3^)	*c*_*L,M*0_ × 10^3^ (kmol/m^3^)	*x*_1_	*x*_2_	*x*_3_	*c*_*IAA,Ff,ex*_ × 10^4^ (kmol/m^3^)	*c*_*IAA,Sf,ex*_ × 10^3^ (kmol/m^3^)	*E*_*F,ex*_ (%)	*K*_*D,ex*_	*E*_*R,ex*_ (%)
1	0.1	10	10	− 1	− 1	− 1	0.07	0.22	93.0	13.3	78.1
2	1	10	10	1	− 1	− 1	1.00	1.90	90.0	9.00	66.5
3	0.1	1000	10	− 1	1	− 1	0.03	0.26	97.0	32.3	91.0
4	1	1000	10	1	1	− 1	0.42	2.58	95.8	22.8	90.2
5	0.1	10	100	− 1	− 1	1	0.04	0.24	96.0	24.0	85.1
6	1	10	100	1	− 1	1	0.50	2.00	95.0	19.0	70.0
7	0.1	1000	100	− 1	1	1	0.02	0.27	97.9	46.6	94.2
8	1	1000	100	1	1	1	0.28	2.37	97.2	34.7	83.0
9	0.55	505	55	0	0	0	0.25	1.11	95.5	21.3	70.6
10	0.55	505	55	0	0	0	0.26	1.08	95.3	20.3	68.7
11	0.55	505	55	0	0	0	0.23	1.15	95.8	22.8	73.2
12	0.55	505	55	0	0	0	0.24	1.13	95.7	22.3	71.9

Moreover, 4 centre-point runs (9–12 in Table 1) were performed. Statistical models described by Eq. (7) link the process dimensionless factors, *x*_*j*_ (*j* = 1..3), and their interactions (*x*_1_*x*_2_, *x*_1_*x*_3_, *x*_2_*x*_3_, and *x*_1_*x*_2_*x*_3_) to the process responses, *y*_*i*_ (*i* = 1..5), i.e*.*, *y*_1_ = *c*_*IAA,Ff*_ × 10^4^, *y*_2_ = *c*_*IAA,Sf*_ × 10^3^, *y*_3_ = *E*_*F*_, *y*_4_ = *K*_*D*_, and *y*_5_ = *E*_*R*_. Regression coefficients, *β*_*ki*_ (*k* = 1..8, *i* = 1..5), were determined based on experimental data summarized in Table 1.

7 $$y_{i} = \beta_{1i} + \beta_{2i} x_{1} + \beta_{3i} x_{2} + \beta_{4i} x_{3} + \beta_{5i} x_{1} x_{{2}} + \beta_{6i} x_{1} x_{{3}} + \beta_{7i} x_{2} x_{{3}} + \beta_{8i} x_{1} x_{{2}} x_{3}$$

#### Mass transfer-based model

Some characteristic parameters of mass transfer process in the three-phase system, i.e., association constants in membrane phase (*K*_*as,M*_) and feed solution (*K*_*as,F*_), repartition constants of species *IAA* and *L* (*TOA*) between membrane and feed solution (*R*_*IAA,M/F*_ and *R*_*L,M/F*_), repartition constant of species *L* between membrane and stripping solution (*R*_*L,M/S*_), and extraction constant (*K*_*ext*_), are defined by Eqs. (8)–(11), where *c*_*s,p*_ (kmol/m^3^) represents the molar concentration of *s* (*IAA*, *L*, *IAA-L*) species in the *p* (*F*, *M*, *S*) phase^32^.

8 $$K_{as,p} = \frac{{c_{IAA - L,p} }}{{c_{IAA,p} c_{L,p} }},p = F,M$$

9 $$R_{IAA,M/F} = \frac{{c_{IAA,M} }}{{c_{IAA,F} }}$$

10 $$R_{L,M/p} = \frac{{c_{L,M} }}{{c_{L,p} }},p = F,S$$

11 $$K_{ext} = \frac{{c_{IAA - L,M} }}{{c_{IAA,F} c_{L,M} }}$$

The partial mass balance of *L* species in the three-phase system, considering perfectly mixed phases, is given by Eq. (12), where *V*_*p*_ (m^3^) is the volume of *p* (*F*, *M*, *S*) phase and *c*_*L,M*0_ (kmol/m^3^) the initial molar concentration of *L* species in the membrane phase. Dividing the terms of Eq. (12) to *V*_*M*_, Eq. (13) was obtained, where *r*_*M/p*_ (*p* = *F*, *S*) volume ratios are defined by Eq. (14).

12 $$c_{L,M0} V_{M} = \left( {c_{L,M} + c_{IAA - L,M} } \right)V_{M} + \left( {c_{L,F} + c_{IAA - L,F} } \right)V_{F} + c_{L,S} V_{S}$$

13 $$c_{L,M0} = \left( {c_{L,M} + c_{IAA - L,M} } \right) + \frac{{\left( {c_{L,F} + c_{IAA - L,F} } \right)}}{{r_{M/F} }} + \frac{{c_{L,S} }}{{r_{M/S} }}$$

14 $$r_{M/p} = \frac{{V_{M} }}{{V_{p} }},p = F,S$$

Substituting Eqs. (8)–(11) into Eq. (13), *c*_*L,M*0_ is given by Eq. (15), where *H* is defined by Eq. (16). Assuming *H*≈1 ($R_{L,M/F} r_{M/F}$»1 and $R_{L,M/S} r_{M/S}$»1), *c*_*IAA-L,M*_ can be expressed depending on *c*_*L,M*0_, *c*_*IAA,F*_, and *K*_*ext*_ using Eq. (17).

15 $$c_{L,M0} = c_{IAA - L,M} \left( {1 + \frac{H}{{K_{ext} c_{IAA,F} }}} \right)$$

16 $$H = 1 + \frac{{K_{as,F} c_{IAA,F} }}{{R_{L,M/F} r_{M/F} }} + \frac{1}{{R_{L,M/F} r_{M/F} }} + \frac{1}{{R_{L,M/S} r_{M/S} }}$$

17 $$c_{IAA - L,M} = \frac{{c_{L,M0} }}{{1 + \frac{1}{{K_{ext} c_{IAA,F} }}}}$$

Total molar flux of *IAA* (*J*_*IAA,tot*_) is defined by Eq. (18) as sum of molar flux of free *IAA* (*J*_*IAA*_) and molar flux of associated *IAA* (*J*_*IAA-L*_). Assuming *J*_*IAA*_«*J*_*IAA-L*_, *J*_*IAA,tot*_ [kmol/(m^2^·s)] can be expressed by Eq. (19), where *k*_*IAA-L,M*_ (m/s) represents the mass transfer coefficient of *IAA-L* complex in the membrane phase. Substituting Eq. (17) into Eq. (19) and considering a stationary state characterized by a mean flux *J*_*IAA,tot,m*_, Eq. (20) was obtained, where *c*_*IAA,F,m*_ is a mean concentration of *IAA* in the feed phase. According to Eq. (20), *k*_*IAA-L,M*_ and *K*_*ext*_ can be estimated from the intercept and slope of the straight line given by a plot of $\frac{{c_{L,M0} }}{{J_{IAA,tot,m} }}$ *vs.* $\frac{1}{{c_{IAA,F,m} }}$. The parameters *k*_*IAA-L,M*_ and *K*_*ext*_ were determined based on experimental data obtained in 16 experimental runs (1–8 and 13–20 in Table 2), which were performed at different levels of *c*_*IAA,F*0_ (0.0001, 0.0003, 0.0006, and 0.001 kmol/m^3^), *c*_*NaOH,S*0_ (0.01 and 1 kmol/m^3^), and *c*_*L,M*0_ (0.01 and 0.1 kmol/m^3^).

18 $$J_{IAA,tot} = J_{IAA} + J_{IAA - L}$$

19 $$J_{IAA,tot} \approx J_{IAA - L} = k_{IAA - L,M} c_{IAA - L,M}$$

20 $$\frac{{c_{L,M0} }}{{J_{IAA,tot,m} }} = \frac{1}{{k_{IAA - L,M} }} + \frac{1}{{k_{IAA - L,M} K_{ext} }}\frac{1}{{c_{IAA,F,m} }}$$

**Table 2:** Values of final molar concentration of *IAA* in the stripping solution, mean logarithmic concentration of *IAA* in the feed phase, and mean total flux of *IAA* at different levels of process factors.

No.	Run	*c*_*IAA,F*0_ × 10^3^ (kmol/m^3^)	*c*_*NaOH,S*0_ × 10^3^ (kmol/m^3^)	*c*_*L,M*0_ × 10^3^ (kmol/m^3^)	*c*_*IAA,Sf,ex*_ × 10^3^ (kmol/m^3^)	*c*_*IAA,F,m*_ × 10^3^ (kmol/m^3^)	*J*_*IAA,tot,m*_ × 10^9^ [kmol/(m^2^·s)]
1	1	0.1	10	10	0.22	0.035	0.382
2	13	0.3	10	10	0.63	0.109	1.087
3	14	0.6	10	10	1.20	0.223	2.057
4	2	1	10	10	1.90	0.391	3.258
5	3	0.1	1000	10	0.26	0.038	0.446
6	15	0.3	1000	10	0.75	0.128	1.279
7	16	0.6	1000	10	1.42	0.281	2.439
8	4	1	1000	10	2.58	0.388	4.418
9	5	0.1	10	100	0.24	0.045	0.416
10	17	0.3	10	100	0.69	0.146	1.190
11	18	0.6	10	100	1.30	0.320	2.234
12	6	1	10	100	2.00	0.581	3.429
13	7	0.1	1000	100	0.27	0.033	0.460
14	19	0.3	1000	100	0.77	0.117	1.323
15	20	0.6	1000	100	1.51	0.249	2.586
16	8	1	1000	100	2.37	0.468	4.066

## Results and discussions

### Statistical models

The values of dimensional and dimensionless factors and those of process responses determined experimentally, i.e*.*, *c*_*IAA,Ff,ex*_ = 0.02–1 × 10^–4^ kmol/m^3^, *c*_*IAA,Sf,ex*_ = 0.22–2.58 × 10^–3^ kmol/m^3^, *E*_*F,ex*_ = 90.0–97.9%, *K*_*D,ex*_ = 9.0–46.6, and *E*_*R,ex*_ = 66.5–94.2%, are summarized in Table 1. Tabulated data (runs 1–8) highlight the following issues: (1) lower values of *c*_*IAA,Ff,ex*_ at inferior levels of *c*_*IAA,F*0_ and superior levels of *c*_*NaOH,S*0_ and *c*_*L,M*0_, the effect of *c*_*IAA,F*0_ being significant (12.5–14.3 times); (2) higher values of *c*_*IAA,Sf,ex*_ at superior levels of *c*_*IAA,F*0_ and *c*_*NaOH,S*0_, the effect of *c*_*IAA,F*0_ being significant (8.2–9.9 times); (3) higher values of *E*_*F,ex*_ and *K*_*D,ex*_ at inferior level of *c*_*IAA,F*0_ and superior levels of *c*_*NaOH,S*0_ and *c*_*L,M*0_; (4) higher values of *E*_*R,ex*_ at inferior level of *c*_*IAA,F*0_, superior level of *c*_*NaOH,S*0_, and, except for runs 4 and 8, at superior level of *c*_*L,M*0_.

An increase in extraction and recovery efficiencies and distribution coefficient with an increase in *c*_*L,M*0_ and *c*_*NaOH,S*0_ was reported in the related literature^10,11,13,18,25,27,30^. A higher level of *c*_*L,M*0_ leads to an increase in the concentration of *IAA-L* complex in the membrane phase, resulting in enhanced mass transfer of this complex. On the other hand, a higher value of *c*_*NaOH,S*0_ determines enhanced mass transfer of *IAA-L* complex by increasing the concentration of *L* released in the membrane phase after decomplexation^10,11,13^. Moreover, a decrease in extraction and recovery efficiencies with an increase in *c*_*IAA,F*0_ was found by other researchers^18^.

Statistical models given by Eqs. (21)–(25) express the process responses depending on dimensionless factors and their interactions. Regression coefficients, *β*_*ki*_ (*k* = 1..8, *i* = 1..5), which were determined by processing the experimental data presented in Table 1, are summarized in Supplementary Tables S1–S5 along with their corresponding values of standard errors (*SE*_*ki*_), *t* statistics (*t*_*ki*_), and *p*-values (*p*_*ki*_). The coefficients that are statistically significant (*p*_*ki*_ ≤ *α* = 0.05, where *α* is the significance level) are written in bold. Supplementary Tables S1–S5 contain also the values of multiple determination coefficient (*R*^2^), adjusted *R*^2^ (*R*^2^_*adj*_), regression standard error (*RSE*), *F* statistic (*F*), and significance *F* (*p*-value for *F*).

Tabulated results indicate that Eqs. (21)–(24) fit the data very well (*R*^2^ ≥ 0.966, *R*^2^_*adj*_ ≥ 0.906, *RSE* ≤ 3.050, *F* ≥ 16.13, *p* ≤ 0.009), whereas Eq. (25) does not fit the data well (*R*^2^ = 0.674, *R*^2^_*adj*_ = 0.104, *RSE* = 9.233, *F* = 1.182, *p* = 0.462). Moreover, all factors and their binary and ternary interactions in Eq. (25) (Supplementary Table S5) are statistically non-significant, i.e., *p*_*k*5_ > 0.05 (*k* = 2..8). Quadratic regression Eq. (26), where *β*_*k*5_ (*k* = 1..10) are regression coefficients, was selected to express *y*_5_ = *E*_*R*_. The results of multiple regression analysis, which are given in Supplementary Table S6, highlight that the model is statistically significant (*F* = 35.20, *p* = 0.028). Moreover, *R*^2^ = 0.984, *R*^2^_*adj*_ = 0.456, *RSE* = 2.044 as well as *x*_1_, *x*_2_, and *x*_2_^2^ have statistically significant effects on *E*_*R*_.

21 $$y_{1} = c_{IAA,Ff} \times 10^{4} = 0.278 + 0.255x_{1} - 0.107x_{2} - 0.085x_{{3}} - 0.093x_{1} x_{{2}} - 0.075x_{1} x_{{3}} + 0.048x_{2} x_{{3}} + 0.042x_{1} x_{{2}} x_{3}$$

22 $$y_{2} = c_{IAA,Sf} \times 10^{3} = 1.193 + 0.982x_{1} + 0.139x_{2} - 0.010x_{{3}} + 0.123x_{1} x_{{2}} - 0.017x_{1} x_{{3}} - 0.040x_{2} x_{{3}} - 0.037x_{1} x_{{2}} x_{3}$$

23 $$y_{3} = E_{F} = 95.35 - 0.737x_{1} + 1.738x_{2} + 1.288x_{{3}} + 0.262x_{1} x_{{2}} + 0.313x_{1} x_{{3}} - 0.712x_{2} x_{{3}} - 0.188x_{1} x_{{2}} x_{3}$$

24 $$y_{4} = K_{D} = 24.04 - 3.839x_{1} + 8.899x_{2} + 5.863x_{{3}} - 1.518x_{1} x_{{2}} - 0.387x_{1} x_{{3}} + 0.685x_{2} x_{{3}} - 0.208x_{1} x_{{2}} x_{3}$$

25 $$y_{5} = E_{R} = 78.53 - 4.821x_{1} + 7.341x_{2} + 0.805x_{{3}} + 1.829x_{1} x_{{2}} - 1.733x_{1} x_{{3}} - 1.820x_{2} x_{{3}} - 0.834x_{1} x_{{2}} x_{3}$$

26 $$y_{5} = E_{R} = \beta_{15} + \beta_{25} x_{1} + \beta_{35} x_{2} + \beta_{45} x_{3} + \beta_{55} x_{1} x_{{2}} + \beta_{65} x_{1} x_{{3}} + \beta_{75} x_{2} x_{{3}} + \beta_{85} x_{1}^{2} + \beta_{95} x_{2}^{2} + \beta_{105} x_{3}^{2}$$

Statistical models expressed by Eq. (21) and Eqs. (27)–(30), obtained after removing statistically non-significant terms in Eqs. (22)–(24) and (26), along with their corresponding results of multiple regression analysis (Supplementary Tables S1, S7–S10) indicate the following aspects: (1) *c*_*IAA,Ff*_ decreases with a decrease in *x*_1_, *x*_2_*x*_3_, and *x*_1_*x*_2_*x*_3_ as well as with an increase in *x*_2_, *x*_3_, *x*_1_*x*_2_, and *x*_1_*x*_3_; (2) *c*_*IAA,Sf*_ increases with an increase in *x*_1_, *x*_2_, and *x*_1_*x*_2_; (3) *E*_*F*_ increases with a decrease in *x*_1_ and *x*_2_*x*_3_ as well as with an increase in *x*_2_ and *x*_3_; (4) higher levels of *K*_*D*_ correspond to lower values of *x*_1_ and higher values of *x*_2_ and *x*_3_; (5) higher levels of *E*_*R*_ correspond to lower values of *x*_1_ and higher values of *x*_2_ and *x*_2_^2^; (6) *x*_1_ has the most significant influence on *c*_*IAA,Ff*_ and *c*_*IAA,Sf*_, whereas *x*_2_ has a major effect on *E*_*F*_, *K*_*D*_, and *E*_*R*_; (7) Eq. (21) and Eqs. (27)–(30) fit the data very well (*R*^2^ ≥ 0.905, *R*^2^_*adj*_ ≥ 0.870, *RSE* ≤ 3.521, *F* ≥ 25.47, *p* ≤ 1.9E-04).

27 $$y_{2} = c_{IAA,Sf} \times 10^{3} = 1.193 + 0.982x_{1} + 0.139x_{2} + 0.123x_{1} x_{{2}}$$

28 $$y_{3} = E_{F} = 95.35 - 0.737x_{1} + 1.738x_{2} + 1.288x_{{3}} - 0.712x_{2} x_{{3}}$$

29 $$y_{4} = K_{D} = 24.04 - 3.839x_{1} + 8.899x_{2} + 5.863x_{{3}}$$

30 $$y_{5} = E_{R} = 71.11 - 4.821x_{1} + 7.341x_{2} + 11.13x_{2}^{2}$$

Regression Eqs. (21) and (27)–(30) could be applied to estimate the process performances for factor levels within the ranges considered in the statistical analysis, i.e*.*, *c*_*IAA,F*0_ = 10^–4^–10^–3^ kmol/m^3^, *c*_*NaOH,S*0_ = 10^–2^–1 kmol/m^3^, and *c*_*L,M*0_ = 10^–2^–10^–1^ kmol/m^3^.

Correlation coefficients (*r*) summarized in Supplementary Table S11 indicate: (1) very strong positive correlation between *E*_*F*_ and *K*_*D*_ (*r* = 0.95); (2) strong positive correlations between *c*_*IAA,Ff*_ and *c*_*IAA,Sf*_ (*r* = 0.70), *K*_*D*_ and *E*_*R*_ (*r* = 0.68), and *E*_*F*_ and *E*_*R*_ (*r* = 0.63); (3) strong negative correlations between *c*_*IAA,Ff*_ and *E*_*F*_ (*r* = − 0.68), *c*_*IAA,Ff*_ and *K*_*D*_ (*r* = − 0.67), and *c*_*IAA,Ff*_ and *E*_*R*_ (*r* = − 0.58).

### Mass transfer-based model

The values of *c*_*IAA,Sf,ex*_, *c*_*IAA,F,m*_, and *J*_*IAA,tot,m*_ at different levels of *c*_*IAA,F*0_ (0.0001, 0.0003, 0.0006, and 0.001 kmol/m^3^), *c*_*NaOH,S*0_ (0.01 and 1 kmol/m^3^), and *c*_*L,M*0_ (0.01 and 0.1 kmol/m^3^) are presented in Table 2. The mean logarithmic concentration of *IAA* in the feed phase, *c*_*IAA,F,m*_, and mean total flux of *IAA*, *J*_*IAA,tot,m*_, were calculated using Eqs. (31) and (32). Moreover, *c*_*IAA,Ff*_ and *c*_*IAA,Sf*_ predicted by Eqs. (21) and (22) can be used in Eqs. (31) and (32) instead of *c*_*IAA,Ff,ex*_ and *c*_*IAA,Sf,ex*_. Equation (32) highlights that *J*_*IAA,tot,m*_ [kmol/(m^2^·s)] is proportional with *c*_*IAA,Sf*_ (kmol/m^3^), i.e*.*, *J*_*IAA,tot,m*_ = 1.71 × 10^−6^*c*_*IAA,Sf*_. Accordingly, taking into account Eq. (22), *x*_1_ (dimensionless *c*_*IAA,F*0_), *x*_2_ (dimensionless *c*_*NaOH,S*0_), and their interaction (*x*_1_*x*_2_) have positive effects on process kinetics (evaluated as *J*_*IAA,tot,m*_), the effect of *x*_1_ being higher. The values of *J*_*IAA,tot,m*_ specified in Table 2, i.e*.*, 0.382–4.418 × 10^–9^ kmol/(m^2^·s), are consistent with those estimated in other related studies^17,37^.

31 $$c_{IAA,F,m} = \frac{{c_{IAA,F0} - c_{IAA,Ff,ex} }}{{\ln \left( {\frac{{c_{IAA,F0} }}{{c_{IAA,Ff,ex} }}} \right)}}$$

32 $$J_{IAA,tot,m} = 4\frac{{c_{IAA,Sf,ex} V_{S} }}{{\pi d_{in}^{2} \tau_{f} }}$$

**Figure 2:**
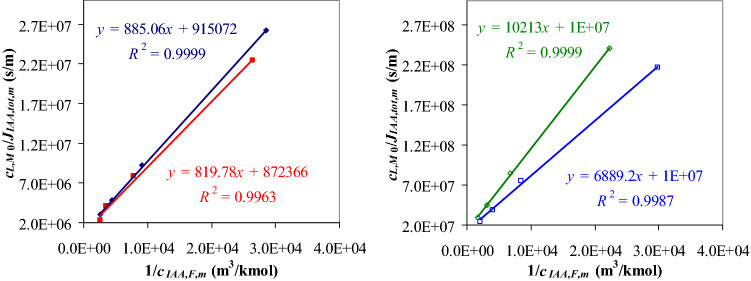
Variation of *c*_*L,M*0_/*J*_*IAA,tot,m*_ depending on 1/*c*_*IAA,F,m*_ under different operating conditions: (filled diamond) *c*_*L,M*0_ = 0.01 kmol/m^3^, *c*_*NaOH,S*0_ = 0.01 kmol/m^3^; (filled square) *c*_*L,M*0_ = 0.01 kmol/m^3^, *c*_*NaOH,S*0_ = 1 kmol/m^3^; (white diamond) *c*_*L,M*0_ = 0.1 kmol/m^3^, *c*_*NaOH,S*0_ = 0.01 kmol/m^3^; (white square) *c*_*L,M*0_ = 0.1 kmol/m^3^, *c*_*NaOH,S*0_ = 1 kmol/m^3^ (*c*_*IAA,F*0_ = 10^–4^–10^–3^ kmol/m^3^, *τ*_*f*_ = 14,400 s).

According to Eq. (20), the values of mass transfer coefficient of *IAA-L* complex in the liquid membrane, *k*_*IAA-L,M*_ = 0.82–11.5 × 10^–7^ m/s, and extraction constant, *K*_*ext*_ = 1033.91–1779.66 m^3^/kmol, were obtained from the intercepts and the slopes of the straight lines given by the plots of *c*_*L,M*0_/*J*_*IAA,tot,m*_* vs.* 1/*c*_*IAA,F,m*_ (Fig. 2). The levels of *k*_*IAA-L,M*_ and *K*_*ext*_, which are summarized in Table 3, indicate the following issues: (1) *k*_*IAA-L,M*_ increases with an increase in *c*_*NaOH,S*0_ (up to 5%) and decreases with an increase in *c*_*L,M*0_ (about 14 times); (2) *K*_*ext*_ increases with an increase in *c*_*NaOH,S*0_ (up to 1.4 times) and *c*_*L,M*0_ (up to 1.7 times). The effect of *x*_2_ and *x*_3_ (dimensionless *c*_*NaOH,S*0_ and *c*_*L,M*0_) on mass transfer coefficient and extraction constant can be predicted using Eqs. (33) and (34) (*R*^2^ = 1, *RSE* = 0), obtained based on data given in Table 3. Multiple regression Eq. (34) indicates an increase in extraction constant with an increase in *x*_2_, *x*_3_, and *x*_2_*x*_3_, the effect of *x*_3_ being higher. Multiple regression Eq. (33) highlights that the effect of *x*_3_ on *y* = *k*_*IAA-L,M,calc*_ is over 30 times higher than the effects of *x*_2_ and *x*_2_*x*_3_. Equation (35) was obtained by neglecting the contributions of *x*_2_ and *x*_2_*x*_3_ in Eq. (33). Results specified in Supplementary Table S12 indicate that Eq. (35) fits very well the data presented in Table 3 (*R*^2^ = 0.998, *R*^2^_*adj*_ = 0.998, *RSE* = 0.300, *F* = 1198.4, *p* = 8.3E−04). According to Eq. (35), the mass transfer coefficient is higher at lower levels of initial molar concentration of *TOA* in the membrane phase.

33 $$y = k_{IAA - L,M,calc} \times 10^{7} = 6.0 + 0.158x_{2} - 5.2x_{3} - 0.142x_{2} x_{{3}}$$

34 $$K_{ext,calc} = 1281.69 + 140.21x_{2} + 232.67x_{3} + 125.09x_{2} x_{{3}}$$

35 $$y = 6.0 - 5.2x_{3}$$

**Table 3:** Values of mass transfer coefficient of *IAA-L* complex in the liquid membrane and extraction constant at different levels of process factors.

*c*_*IAA,F*0_ (kmol/m^3^)	*c*_*NaOH,S*0_ (kmol/m^3^)	*c*_*L,M*0_ (kmol/m^3^)	*x*_2_	*x*_3_	*k*_*IAA-L,M*_ × 10^7^ (m/s)	*K*_*ext*_ (m^3^/kmol)
10^–4^–10^–3^	0.01	0.01	− 1	− 1	10.90	1033.91
10^–4^–10^–3^	1	0.01	1	− 1	11.50	1064.14
10^–4^–10^–3^	0.01	0.1	− 1	1	0.784	1249.06
10^–4^–10^–3^	1	0.1	1	1	0.816	1779.66

## Conclusions

*IAA* transport through a chloroform BLM using *TOA* as a ligand (*L*) was performed in a tube in tube equipment. The inner tube contained a stripping (*S*) aqueous solution of *NaOH* and the outer tube the membrane (*M*) phase and a feed (*F*) aqueous solution of *IAA*. Twenty experiments were performed at different levels of process factors in terms of initial molar concentrations of *IAA* in the feed phase, *c*_*IAA,F*0_ (10^–4^–10^–3^ kmol/m^3^), of *TOA* in the membrane phase, *c*_*L,M*0_ (10^–2^ and 10^–1^ kmol/m^3^), and of *NaOH* in the stripping phase, *c*_*NaOH,S*0_ (10^–2^ and 1 kmol/m^3^). Each experimental run was conducted for 4 h, at ambient temperature, under mechanical stirring (200 rpm) of inner tube.

The effects of dimensionless factors on process responses, i.e., final values of molar concentration of *IAA* in the feed phase (*c*_*IAA,Ff*_) and stripping solution (*c*_*IAA,Sf*_), extraction efficiency (*E*_*F*_), distribution coefficient (*K*_*D*_), and recovery efficiency (*E*_*R*_), were quantified using statistical models based on a 2^3^ factorial plan. Experimental values of process responses were as follows: *c*_*IAA,Ff,ex*_ = 0.02–1 × 10^–4^ kmol/m^3^, *c*_*IAA,Sf,ex*_ = 0.22–2.58 × 10^–3^ kmol/m^3^, *E*_*F,ex*_ = 90.0–97.9%, *K*_*D,ex*_ = 9.0–46.6, and *E*_*R,ex*_ = 66.5–94.2%. Taking into account the statistically significant factors and their interactions, the results of regression analysis indicated the following aspects: (1) all factors and their binary and ternary interactions influenced *c*_*IAA,Ff*_; (2) *c*_*IAA,Sf*_ increased with an increase in *c*_*IAA,F*0_, *c*_*NaOH,S*0_, and their binary interaction; (3) higher levels of *E*_*F*_ and *K*_*D*_ were obtained at low values of *c*_*IAA,F*0_ and high values of *c*_*L,M*0_ and *c*_*NaOH,S*0_; (4) higher levels of *E*_*R*_ were obtained at low values of *c*_*IAA,F*0_ and high values of *c*_*NaOH,S*0_; (5) *c*_*IAA,F*0_ had the most significant (positive) effect on *c*_*IAA,Ff*_ and *c*_*IAA,Sf*_, whereas *c*_*NaOH,S*0_ had a major (positive) effect on *E*_*F*_, *K*_*D*_, and *E*_*R*_. A very strong positive correlation (*r* = 0.95) was found between *E*_*F*_ and *K*_*D*_.

A deterministic model based on mass transfer of *IAA* in the system containing the BLM was developed and its parameters, i.e*.*, mass transfer coefficient of *IAA-L* complex in the liquid membrane (*k*_*IAA-L,M*_ = 0.82–11.5 × 10^–7^ m/s) and extraction constant (*K*_*ext*_ = 1033.91–1779.66 m^3^/kmol), were regressed from experimental data. The process factors in terms of *c*_*NaOH,S*0_ and *c*_*L,M*0_ had positive effects on *K*_*ext*_, whereas *c*_*L,M*0_ had a major negative effect on *k*_*IAA-L,M*_.

The results obtained in this study indicate that *IAA* was successfully transported through a chloroform BLM using *TOA* as a carrier. Mathematical models developed in the paper could be used to control and optimize the separation of *IAA* in systems containing liquid membranes.

## Supplementary Information

Supplementary Information.
