# Author Correction: Effects of process factors on performances of liquid membrane-based transfer of indole-3-acetic acid

**DOI:** 10.1038/s41598-022-05212-z

**Published:** 2022-01-14

**Authors:** Ioana Diaconu, Oana Cristina Pârvulescu, Sorina Laura Topală, Tănase Dobre

**Affiliations:** 1 Department of Analytical Chemistry and Environmental Engineering, University POLITEHNICA of Bucharest, 1-6 Gheorghe Polizu, 011061 Bucharest, Romania; 2 Department of Chemical and Biochemical Engineering, University POLITEHNICA of Bucharest, 1-6 Gheorghe Polizu, 011061 Bucharest, Romania

Correction to: *Scientific Reports* 10.1038/s41598-021-02876-x, published online 06 December 2021.

The original version of this Article contained an error.

In the Materials and methods section, under the subheading ‘Statistical models’,

“Moreover, 4 centre-point runs (9–12 in Table 1) were performed. Statistical models described by Eq. (7) link the process dimensionless factors, *x*_*j*_ (*j* = 1.3), and their interactions (*x*_1_*x*_2_, *x*_1_*x*_3_, *x*_2_*x*_3_, and *x*_1_*x*_2_*x*_3_) to the process responses, *y*_*i*_ (*i* = 1.5), i.e*.*, *y*_1_ = *c*_*IAA,Ff*_ × 10^4^, *y*_2_ = *c*_*IAA,Sf*_ × 10^3^, *y*_3_ = *E*_*F*_, *y*_4_ = *K*_*D*_, and *y*_5_ = *E*_*R*_. Regression coefficients, *β*_*ki*_ (*k* = 1.8, *i* = 1.5), were determined based on experimental data summarized in Table 1.”

now reads:

“Moreover, 4 centre-point runs (9–12 in Table 1) were performed. Statistical models described by Eq. (7) link the process dimensionless factors, *x*_*j*_ (*j* = 1..3), and their interactions (*x*_1_*x*_2_, *x*_1_*x*_3_, *x*_2_*x*_3_, and *x*_1_*x*_2_*x*_3_) to the process responses, *y*_*i*_ (*i* = 1..5), i.e*.*, *y*_1_ = *c*_*IAA,Ff*_ × 10^4^, *y*_2_ = *c*_*IAA,Sf*_ × 10^3^, *y*_3_ = *E*_*F*_, *y*_4_ = *K*_*D*_, and *y*_5_ = *E*_*R*_. Regression coefficients, *β*_*ki*_ (*k* = 1..8, *i* = 1..5), were determined based on experimental data summarized in Table 1.”

In addition, in the Results and discussion section, under the subheading ‘Statistical models’,

“Statistical models given by Eqs. (21)–(25) express the process responses depending on dimensionless factors and their interactions. Regression coefficients, *β*_*ki*_ (*k* = 1.8, *i* = 1.5), which were determined by processing the experimental data presented in Table 1, are summarized in Supplementary Tables S1–S5 along with their corresponding values of standard errors (*SE*_*ki*_), *t* statistics (*t*_*ki*_), and *p*-values (*p*_*ki*_).”

now reads:

“Statistical models given by Eqs. (21)–(25) express the process responses depending on dimensionless factors and their interactions. Regression coefficients, *β*_*ki*_ (*k* = 1..8, *i* = 1..5), which were determined by processing the experimental data presented in Table 1, are summarized in Supplementary Tables S1–S5 along with their corresponding values of standard errors (*SE*_*ki*_), *t* statistics (*t*_*ki*_), and *p*-values (*p*_*ki*_).”

“Moreover, all factors and their binary and ternary interactions in Eq. (25) (Supplementary Table S5) are statistically non-significant, i.e*.*, *p*_*k*5_ > 0.05 (*k* = 2.8). Quadratic regression Eq. (26), where *β*_*k*5_ (*k* = 1.10) are regression coefficients, was selected to express *y*_5_ = *E*_*R*_.”

now reads:

“Moreover, all factors and their binary and ternary interactions in Eq. (25) (Supplementary Table S5) are statistically non-significant, i.e*.*, *p*_*k*5_ > 0.05 (*k* = 2..8). Quadratic regression Eq. (26), where *β*_*k*5_ (*k* = 1..10) are regression coefficients, was selected to express *y*_5_ = *E*_*R*_.”

The original Article has been corrected.

