# Supplementary material for: Effects of process factors on performances of liquid membrane-based transfer of indole-3-acetic acid
Source: Sci Rep. 2021 Dec 6;11:23427. doi: 10.1038/s41598-021-02876-x (PMC8648829; doi:10.1038/s41598-021-02876-x)
Supplement: Supplementary file 1 — Supplementary Information. [file 41598_2021_2876_MOESM1_ESM.doc]

**Effects of process factors on performances of liquid membrane-based transfer of indole-3-acetic acid**

(Supplementary information)

**Ioana Diaconu^1^, Oana Cristina Pârvulescu^2*^, Sorina Laura Topală^2^, Tănase Dobre^2^**

^1^University POLITEHNICA of Bucharest, Department of Analytical Chemistry and Environmental Engineering, 1-6 Gheorghe Polizu, 011061, Bucharest, Romania

^2^University POLITEHNICA of Bucharest, Department of Chemical and Biochemical Engineering, 1-6 Gheorghe Polizu, 011061, Bucharest, Romania

**^*^**Corresponding author. E-mail address: [oana.parvulescu@yahoo.com](mailto:oana.parvulescu@yahoo.com).

**Supplementary (S) tables**

| Regressor | *k* | *β_k_*_1_ | *SE_k_*_1_ | *t_k_*_1_ | *p_k_*_1_ |
| --- | --- | --- | --- | --- | --- |
| Intercept | 1 | **0.278** | 0.013 | 21.96 | 3E-05 |
| *x*_1_ | 2 | **0.255** | 0.015 | 16.45 | 8E-05 |
| *x*_2_ | 3 | **-0.107** | 0.015 | -6.931 | 0.002 |
| *x*_3_ | 4 | **-0.085** | 0.015 | -5.479 | 0.005 |
| *x*_1_ *x*_2_ | 5 | **-0.093** | 0.015 | -5.979 | 0.004 |
| *x*_1_ *x*_3_ | 6 | **-0.075** | 0.015 | -4.849 | 0.008 |
| *x*_2_ *x*_3_ | 7 | **0.048** | 0.015 | 3.074 | 0.037 |
| *x*_1_*x*_2_*x*_3_ | 8 | **0.042** | 0.015 | 2.735 | 0.050 |
| *R*^2^ | 0.991 | | | | |
| *R*^2^*_adj_* | 0.974 | | | | |
| *RSE* | 0.044 | | | | |
| *F* | 60.71 | | | | |
| *p* (significance *F*) | 6.7E-04 | | | | |

**Table S1.** Results of multiple regression analysis for *y*_1_ expressed by Eq. (21).

| Regressor | *k* | *β_k_*_2_ | *SE_k_*_2_ | *t_k_*_2_ | *p_k_*_2_ |
| --- | --- | --- | --- | --- | --- |
| Intercept | 1 | **1.193** | 0.028 | 43.16 | 2E-06 |
| *x*_1_ | 2 | **0.982** | 0.034 | 29.01 | 8E-06 |
| *x*_2_ | 3 | **0.139** | 0.034 | 4.104 | 0.015 |
| *x*_3_ | 4 | -0.010 | 0.034 | -0.284 | 0.790 |
| *x*_1_ *x*_2_ | 5 | **0.123** | 0.034 | 3.638 | 0.022 |
| *x*_1_ *x*_3_ | 6 | -0.017 | 0.034 | -0.499 | 0.644 |
| *x*_2_ *x*_3_ | 7 | -0.040 | 0.034 | -1.171 | 0.307 |
| *x*_1_*x*_2_*x*_3_ | 8 | -0.037 | 0.034 | -1.090 | 0.337 |
| *R*^2^ | 0.995 | | | | |
| *R*^2^*_adj_* | 0.987 | | | | |
| *RSE* | 0.096 | | | | |
| *F* | 124.9 | | | | |
| *p* (significance *F*) | 1.6E-04 | | | | |

**Table S2.** Results of multiple regression analysis for *y*_2_ expressed by Eq. (22).

| Regressor | *k* | *β_k_*_3_ | *SE_k_*_3_ | *t_k_*_3_ | *p_k_*_3_ |
| --- | --- | --- | --- | --- | --- |
| Intercept | 1 | **95.35** | 0.098 | 974.4 | 7E-12 |
| *x*_1_ | 2 | **-0.737** | 0.120 | -6.154 | 0.004 |
| *x*_2_ | 3 | **1.738** | 0.120 | 14.50 | 1E-04 |
| *x*_3_ | 4 | **1.288** | 0.120 | 10.74 | 4E-04 |
| *x*_1_ *x*_2_ | 5 | 0.262 | 0.120 | 2.190 | 0.094 |
| *x*_1_ *x*_3_ | 6 | 0.313 | 0.120 | 2.608 | 0.060 |
| *x*_2_ *x*_3_ | 7 | **-0.712** | 0.120 | -5.945 | 0.004 |
| *x*_1_*x*_2_*x*_3_ | 8 | -0.188 | 0.120 | -1.565 | 0.193 |
| *R*^2^ | 0.990 | | | | |
| *R*^2^*_adj_* | 0.974 | | | | |
| *RSE* | 0.339 | | | | |
| *F* | 58.98 | | | | |
| *p* (significance *F*) | 7.1E-04 | | | | |

**Table S3.** Results of multiple regression analysis for *y*_3_ expressed by Eq. (23).

| Regressor | *k* | *β_k_*_4_ | *SE_k_*_4_ | *t_k_*_4_ | *p_k_*_4_ |
| --- | --- | --- | --- | --- | --- |
| Intercept | 1 | **24.04** | 0.880 | 27.30 | 1E-05 |
| *x*_1_ | 2 | **-3.839** | 1.078 | -3.560 | 0.024 |
| *x*_2_ | 3 | **8.899** | 1.078 | 8.252 | 0.001 |
| *x*_3_ | 4 | **5.863** | 1.078 | 5.437 | 0.006 |
| *x*_1_ *x*_2_ | 5 | -1.518 | 1.078 | -1.408 | 0.232 |
| *x*_1_ *x*_3_ | 6 | -0.387 | 1.078 | -0.359 | 0.738 |
| *x*_2_ *x*_3_ | 7 | 0.685 | 1.078 | 0.635 | 0.560 |
| *x*_1_*x*_2_*x*_3_ | 8 | -0.208 | 1.078 | -0.193 | 0.856 |
| *R*^2^ | 0.966 | | | | |
| *R*^2^*_adj_* | 0.906 | | | | |
| *RSE* | 3.050 | | | | |
| *F* | 16.13 | | | | |
| *p* (significance *F*) | 0.009 | | | | |

**Table S4.** Results of multiple regression analysis for *y*_4_ expressed by Eq. (24).

| Regressor | *k* | *β_k_*_5_ | *SE_k_*_5_ | *t_k_*_5_ | *p_k_*_5_ |
| --- | --- | --- | --- | --- | --- |
| Intercept | 1 | **78.53** | 2.665 | 29.463 | 8E-06 |
| *x*_1_ | 2 | -4.821 | 3.265 | -1.477 | 0.214 |
| *x*_2_ | 3 | 7.341 | 3.265 | 2.249 | 0.088 |
| *x*_3_ | 4 | 0.805 | 3.265 | 0.247 | 0.817 |
| *x*_1_ *x*_2_ | 5 | 1.829 | 3.265 | 0.560 | 0.605 |
| *x*_1_ *x*_3_ | 6 | -1.733 | 3.265 | -0.531 | 0.624 |
| *x*_2_ *x*_3_ | 7 | -1.820 | 3.265 | -0.558 | 0.607 |
| *x*_1_*x*_2_*x*_3_ | 8 | -0.834 | 3.254 | -0.256 | 0.810 |
| *R*^2^ | 0.674 | | | | |
| *R*^2^*_adj_* | 0.104 | | | | |
| *RSE* | 9.233 | | | | |
| *F* | 1.182 | | | | |
| *p* (significance *F*) | 0.462 | | | | |

**Table S5.** Results of multiple regression analysis for *y*_5_ expressed by Eq. (25).

| Regressor | *k* | *β_k_*_5_ | *SE_k_*_5_ | *t_k_*_5_ | *p_k_*_5_ |
| --- | --- | --- | --- | --- | --- |
| Intercept | 1 | **71.11** | 1.022 | 69.576 | 3.E-07 |
| *x*_1_ | 2 | **-4.821** | 0.723 | -6.671 | 0.003 |
| *x*_2_ | 3 | **7.341** | 0.723 | 10.158 | 0.001 |
| *x*_3_ | 4 | 0.805 | 0.723 | 1.114 | 0.328 |
| *x*_1_ *x*_2_ | 5 | 1.829 | 0.723 | 2.530 | 0.065 |
| *x*_1_ *x*_3_ | 6 | -1.733 | 0.723 | -2.397 | 0.075 |
| *x*_2_ *x*_3_ | 7 | -1.820 | 0.723 | -2.518 | 0.065 |
| *x*_1_^2^ | 8 | 0 | 0 | 65535 | - |
| *x*_2_^2^ | 9 | **11.13** | 1.252 | 8.889 | 0.001 |
| *x*_3_^2^ | 10 | 0 | 0 | 65535 | - |
| *R*^2^ | 0.984 | | | | |
| *R*^2^*_adj_* | 0.456 | | | | |
| *RSE* | 2.044 | | | | |
| *F* | 35.20 | | | | |
| *p* (significance *F*) | 0.028 | | | | |

**Table S6.** Results of multiple regression analysis for *y*_5_ expressed by Eq. (26).

| Regressor | *k* | *β_k_*_2_ | *SE_k_*_2_ | *t_k_*_2_ | *p_k_*_2_ |
| --- | --- | --- | --- | --- | --- |
| Intercept | 1 | **1.193** | 0.026 | 46.52 | 5E-11 |
| *x*_1_ | 2 | **0.982** | 0.031 | 31.26 | 1E-09 |
| *x*_2_ | 3 | **0.139** | 0.031 | 4.423 | 0.002 |
| *x*_1_ *x*_2_ | 5 | **0.123** | 0.031 | 3.921 | 0.004 |
| *R*^2^ | 0.992 | | | | |
| *R*^2^*_adj_* | 0.989 | | | | |
| *RSE* | 0.089 | | | | |
| *F* | 337.4 | | | | |
| *p* (significance *F*) | 9.3E-09 | | | | |

**Table S7.** Results of multiple regression analysis for *y*_2_ expressed by Eq. (27).

| Regressor | *k* | *β_k_*_3_ | *SE_k_*_3_ | *t_k_*_3_ | *p_k_*_3_ |
| --- | --- | --- | --- | --- | --- |
| Intercept | 1 | **95.35** | 0.157 | 606.9 | 8.7E-18 |
| *x*_1_ | 2 | **-0.737** | 0.192 | -3.833 | 6.4E-03 |
| *x*_2_ | 3 | **1.738** | 0.192 | 9.030 | 4.2E-05 |
| *x*_3_ | 4 | **1.288** | 0.192 | 6.691 | 2.8E-04 |
| *x*_2_ *x*_3_ | 7 | **-0.712** | 0.192 | -3.703 | 7.6E-03 |
| *R*^2^ | 0.957 | | | | |
| *R*^2^*_adj_* | 0.932 | | | | |
| *RSE* | 0.544 | | | | |
| *F* | 38.68 | | | | |
| *p* (significance *F*) | 7.3E-05 | | | | |

**Table S8.** Results of multiple regression analysis for *y*_3_ expressed by Eq. (28).

| Regressor | *k* | *β_k_*_4_ | *SE_k_*_4_ | *t_k_*_4_ | *p_k_*_4_ |
| --- | --- | --- | --- | --- | --- |
| Intercept | 1 | **24.04** | 0.797 | 30.17 | 1.6E-09 |
| *x*_1_ | 2 | **-3.839** | 0.976 | -3.935 | 4.3E-03 |
| *x*_2_ | 3 | **8.899** | 0.976 | 9.120 | 1.7E-05 |
| *x*_3_ | 4 | **5.863** | 0.976 | 6.009 | 3.2E-04 |
| *R*^2^ | 0.944 | | | | |
| *R*^2^*_adj_* | 0.923 | | | | |
| *RSE* | 2.760 | | | | |
| *F* | 44.92 | | | | |
| *p* (significance *F*) | 2.4E-05 | | | | |

**Table S9.** Results of multiple regression analysis for *y*_4_ expressed by Eq. (29).

| Regressor | *k* | *β_k_*_5_ | *SE_k_*_5_ | *t_k_*_5_ | *p_k_*_5_ |
| --- | --- | --- | --- | --- | --- |
| Intercept | 1 | **71.11** | 1.760 | 40.40 | 2E-10 |
| *x*_1_ | 2 | **-4.821** | 1.245 | -3.873 | 0.0047 |
| *x*_2_ | 3 | **7.341** | 1.245 | 5.898 | 0.0004 |
| *x*_2_^2^ | 9 | **11.13** | 2.156 | 5.161 | 0.0009 |
| *R*^2^ | 0.905 | | | | |
| *R*^2^*_adj_* | 0.870 | | | | |
| *RSE* | 3.521 | | | | |
| *F* | 25.47 | | | | |
| *p* (significance *F*) | 1.9E-04 | | | | |

**Table S10.** Results of multiple regression analysis for *y*_5_ expressed by Eq. (30).

|  | *c_IAA,Ff_* | *c_IAA,Sf_* | *E_F_* | *K_D_* | *E_R_* |
| --- | --- | --- | --- | --- | --- |
| *c_IAA,Ff_* | 1 |  |  |  |  |
| *c_IAA,Sf_* | **0.70** | 1 |  |  |  |
| *E_F_* | **-0.68** | -0.20 | 1 |  |  |
| *K_D_* | **-0.67** | -0.22 | **0.95** | 1 |  |
| *E_R_* | **-0.58** | -0.34 | **0.63** | **0.68** | 1 |

**Table S11.** Results of correlation analysis [in bold are significant values at *α*=0.05 (two-tailed test)].

| Regressor | *k* | *β_k_* | *SE_k_* | *t_k_* | *p_k_* |
| --- | --- | --- | --- | --- | --- |
| Intercept | 1 | **6.0** | 0.1502 | 39.943 | 6.3E-04 |
| *x*_3_ | 3 | **-5.2** | 0.1502 | -34.617 | 8.3E-04 |
| *R*^2^ | 0.998 | | | | |
| *R*^2^*_adj_* | 0.998 | | | | |
| *RSE* | 0.300 | | | | |
| *F* | 1198.4 | | | | |
| *p* (significance *F*) | 8.3E-04 | | | | |

**Table S12.** Results of multiple regression analysis for *y* expressed by Eq. (35).
